# Social Monitoring Matters for Deterring Social Deviance in Stable but Not Mobile Socio-Ecological Contexts

**DOI:** 10.1371/journal.pone.0167053

**Published:** 2016-11-23

**Authors:** Jenny C. Su, Chi-Yue Chiu, Wei-Fang Lin, Shigehiro Oishi

**Affiliations:** 1 Department of Psychology, St. Lawrence University, Canton, NY, USA; 2 Department of Psychology, Chinese University of Hong Kong, Hong Kong; 3 Department of Psychology, Chung Yuan Christian University, Taoyuan, Taiwan; 4 Department of Psychology, University of Virginia, Charlottesville, VA, USA; University of Texas at San Antonio, UNITED STATES

## Abstract

Previous research suggests that reputational concerns can incentivize cooperation and deter socially deviant behavior. The current research showed that social monitoring of information that has the potential to damage one’s reputation has differential effects on deviant behavior in social-ecological environments that vary in level of mobility. Study 1 showed that residentially stable cities that employed more journalists—who can be regarded as social monitoring agents in a community—tended to have lower rates of violent crime than residentially stable cities that employed fewer journalists; by contrast, in residentially mobile cities, violent crime rates did not vary as a function of the number of journalists employed. In Study 2, we found that individual differences in perceptions of relational mobility moderated the effects of social monitoring on cheating in a die-under-cup game. Specifically, social monitoring cues reduced the likelihood of cheating but only among participants who perceived their immediate social environment to be low in relational mobility. The same results were replicated in Study 3, an experiment in which participants’ perception of relational mobility was manipulated before completing an online maze game that allowed them to earn extra cash. In the low mobility condition, the percentage of participants who continued working on the mazes after reaching the time limit decreased as a function of social monitoring; however, this pattern was not observed in the high mobility condition. Together, our findings suggest that socioecological context matters for understanding effective mechanisms of social control.

## Introduction

Imagine a business executive who helped his company double its sales last year but was given very little bonus for his hard work and personal sacrifice. One day, he was sitting alone in his office deciding whether to use some company funds to pay for a European cruise vacation that he had always wanted to take. Surely, he may lose his job and face the legal consequences of embezzlement if he gets caught. However, the extent to which he would worry about potential damage to his social reputation may depend on how easily he can move around in his social environment.

Over the past decade, a great deal of interest has focused on the role of reputational systems as a viable solution to the problem of cooperation. According to Emler [1], reputation refers to “that set of judgments a community makes about the personal qualities of one of its members” (p. 171). Research has shown that humans have a keen sensitivity to reputational information and are more likely to comply with social norms when they feel that they are being observed by others [2–4]. Indeed, a number of studies have consistently found that reputational concerns can facilitate cooperative behavior and deter people from advancing their own interests at the expense of the common good [5–6].

One explanation for why reputational concerns have such powerful effects on cooperation and deviance is that reputation can influence individuals’ payoff in future social interactions. Studies from evolutionary game theory and economics suggest that people with good reputations are more likely to be selected as interaction partners in future social exchanges [5] and to be helped by others [7–8]. People known to be selfish, on the other hand, are at risk of being socially excluded and even punished [9–10].

Although reputational concerns can incentivize prosociality and deter deviance, the strength of these effects can vary according to socio-ecological constraints [11–12]. Residential mobility and relational mobility are two types of socio-ecological constraints that may influence the effects of reputational concern on pro- and anti-social behaviors. Residential mobility refers to the frequency with which people change residence, whereas relational mobility refers to the number of opportunities individuals have in a given context to voluntarily form new relationships and to terminate old ones [13]. These two concepts have been used to explain a variety of important behavioral and psychological tendencies, including identification with reputable groups and avoidance of negative social reputations (see Oishi et al. [13] for a comprehensive review on residential and relational mobility).

Previous research suggests that reputational information may be more useful in residentially and relationally stable settings where the cost of non-cooperation is much higher. In those settings, there are fewer opportunities to form and reform relationships [14]. Once people are socially excluded for misconduct, they may not be able to find alternative groups or relationships to join. Because relationship networks in less mobile contexts tend to be more stable and close-knit, a stronger emphasis is placed on the collective relative to the individual, as shown by greater commitment to group or community welfare [15] and greater willingness to use punishment against wrongdoings [16].

Scholars have posited that systems of mutual monitoring and sanctioning encourage and sustain a high level of group-based cooperation in collectivistic societies [12, 17]. Monitoring and sharing of reputational information are mechanisms within groups that are designed to detect prosociality and deviance, and are therefore considered useful forms of social control [18–19]. This idea is consistent with empirical evidence showing that accountability and public evaluation enhance cooperative behavior among collectivists [20]. Given the resemblance of the socio-ecological conditions between collectivistic societies and residentially/relationally stable environments [21], monitoring of reputational information is expected to sway individuals toward cooperation and away from misbehavior in contexts that are low in residential or relational mobility. Conversely, monitoring of reputational information may be a less effective means of social control when mobility is high, because the consequences of wrongdoings are less severe as individuals can change social circles to avoid social exclusion and conflict. Past research also indicate that individuals from mobile societies tend to feel less obligated to monitor and respond to dishonest behavior than those from stable societies [16].

Three studies examined whether the usefulness of social monitoring in preventing socially deviant behavior differs according to the given socio-ecological context. Specifically, we tested the hypothesis that the presence of social monitoring will be effective in deterring social deviance when residential or relational mobility is low but not when residential or relational mobility is high. The first study examined these factors at the community level, and the latter two studies examined these factors at the individual level. In Study 1, we examined within-nation, regional variation in crime rate as a function of the number of journalists (social monitoring agents) employed and levels of residential mobility. Given that social monitoring matters more in residentially stable communities, the negative relationship between number of journalists and crime rate should be stronger in those communities than in communities that are residentially mobile. In Study 2, we measured subjective perceptions of relational mobility at the individual level and examined their relationship to lying when a social monitoring cue was either present or absent in the laboratory. In Study 3, we manipulated both individuals’ perception of relational mobility and the presence vs. absence of a social monitoring cue in the laboratory and compared their interactive effects on cheating behaviors.

## Study 1

To test whether the effects of social monitoring are likely to be stronger in residentially stable communities where damages to one’s reputation matter more, we examined crime rates in U.S. cities that differ in rates of journalist employment (an index of the amount of social monitoring that exists in a given community) and levels of residential mobility. The power of the news media to influence the reputation of individuals as well as organizations is well documented [22–23]. By raising public awareness of social problems and threats, social monitoring by journalists may play a role in keeping social deviance under control in residentially stable communities. Based on previous research on relational and residential mobility, we predicted that having more monitoring agents like journalists will have greater effects on crime rates in cities that are low in residential mobility than those that are high in residential mobility.

### Method

We obtained data on the number of reporters and correspondents employed in different U.S. cities from the U.S. Bureau of Labor Statistics (BLS) website (https://www.bls.gov). More specifically, we focused on the number of reporters and correspondents employed per 1,000 jobs in each metropolitan statistical area (MSA). Employment data for reporters and correspondents in 125 MSAs were available for the most recent year (i.e., 2014). For each MSA, we obtained residential mobility data from the U.S. Census Bureau’s American FactFinder website (http://factfinder.census.gov/). More specifically, we used the 5-year estimates of mobility based on data collected from the American Community Survey between 2010 and 2014. Mobility was computed as the total percent of the population (1 year and over) in each MSA that had moved (either within the same state or from a different state) in the past 12 months. Data on violent crime rate per 100,000 inhabitants for 123 of the 125 MSAs were obtained from the Federal Bureau of Investigation’s Uniform Crime Reporting (UCR) website (https://www.fbi.gov/about-us/cjis/ucr/ucr). We decided to focus on violent crimes (i.e., murder and non-negligent manslaughter, rape, robbery, and aggravated assault) in this study, because they are featured most prominently in the media.

Finally, we obtained data on three characteristics of communities that might be confounded with residential stability and crime rate: per capita GDP, unemployment rate, and population size. More specifically, we obtained data on each MSA’s per capita GDP for 2014 from the U.S. Bureau of Economic Analyses (http://www.bea.gov/). In addition, we obtained the 2014 unemployment rate for each MSA from the BLS website. Population size was obtained from the U.S. Federal Bureau of Investigation’s 2014 population estimates, which were derived by adding each MSA’s rate of growth between 2011 and 2013 to its 2013 U.S. Census Bureau population estimate.

### Results

We tested our hypothesis using hierarchical linear regression. The dependent variable, violent crime rate, was transformed using natural logs so that the residuals fit the Gaussian distribution. Results from the Kolmogorov–Smirnov test and the Shapiro–Wilk test indicated that the distribution of the residuals were normal after log transformation (*p*s > .179). To reduce multicollinearity between the predictors, we centered them via z-transformation before creating an interaction term between number of reporters and level of residential mobility. Per capita GDP, unemployment rate, and population size were entered as covariates in Step 1 of the regression. Number of reporters and residential mobility were entered in Step 2. The interaction between number of reporters and level of residential mobility was entered in Step 3.

**Fig 1:**
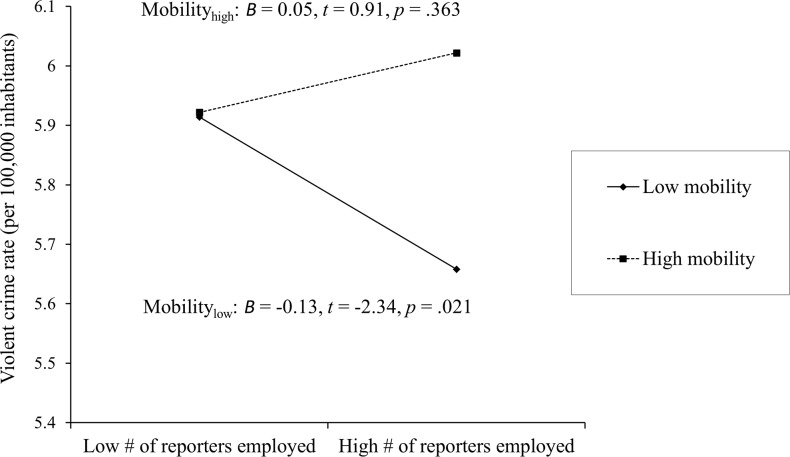
Residential mobility moderates the link between number of reporters employed and violent crime rate.

The regression results suggest that unemployment rate and residential mobility predicted violent crime rate (unemployment rate: *B* = .13, *SE* = .04, *t* = 3.25, *p* = .002, 95% CI = [.052, .214]; residential mobility: *B* = .09, *SE* = .04, *t* = 2.39, *p* = .018, 95% CI = [.016, .169]). Consistent with our hypothesis, we found an interaction between number of reporters and level of residential mobility. The overall model explained 14% of the variance in violent crime rate, *R*^2^ = .14, *F*(6, 116) = 3.16, *p* = .007. The interaction explained an additional 3.1% of the variance in violent crime rate beyond the first-order effects (_Δ_*R*^2^ = .03, *p* = .041) and yielded a moderate effect size (*f*^2^ = .16). As seen in Fig 1, in cities that are low in residential mobility, number of reporters was associated with lower rates of violent crime. In cities that are high in residential mobility, violent crime rate did not change as a function of number of reporters employed.

## Study 2

The main goal of Study 2 (and Study 3) was to conceptually replicate Study 1 by examining the same factors at the individual level. Although we found the expected moderating effect of residential mobility in the relation between social monitoring and deviant behavior, these findings were at the level of city, not individuals. To avoid the ecological fallacy, therefore, it is important to test whether the observed interaction could be replicated at the level of individuals [24]. In addition, violent crime is a relatively infrequent deviant behavior. Thus, it is important to test our hypothesis with more common behaviors. We conducted Study 2 to address these limitations. Specifically, we manipulated the presence vs. absence of social monitoring cues in the laboratory using a surveillance camera and examined whether it would have differential effects on cheating depending on individual variations in subjective perceptions of relational mobility. The presence of a camera is an established and well-validated cue that has been found to induce public self-consciousness [25, 26]. To measure cheating, we used the die-under-cup task, which is a procedure that has previously been used to detect cheating at the individual level [27–28].

### Method

#### Participants

Sixty-four students (38 females, 26 males; average age = 20.30 years) from National Taiwan University (NTU) participated in the study for course credit. Previous studies on this topic yielded effect sizes with a mean *d* of 1.09 (*d*s of .485, .630, and 2.177 were found in Bateson et al. [2], Burnham & Hare [29], and Haley & Fessler [4], respectively). Given that the target effect is an unknown interaction between social monitoring and mobility, we chose an effect size estimate that is more conservative than the mean. With this effect size estimate, *d* = .630, we determined that 82 participants would be needed to have 80% power and thus recruited 83 participants by the end of the semester. However, due to concern over language comprehension of the instructions, we included only participants who were born and raised in Taiwan (15 were excluded for this reason). Additionally, we excluded three participants who failed to answer validity check items correctly and one participant whose score on the relational mobility scale was 3.53 standard deviations below the mean, yielding a final sample size of 64.

#### Procedure

The study comprised of two phases. During the first phase, participants completed an online survey containing the Relational Mobility Scale [30] and basic demographic questions (e.g., age, gender, father’s and mother’s education, and family income). The Relational Mobility Scale was comprised of 12 items that measured individuals’ perception of relational mobility in their immediate social environment. The items were rated using a six-point Likert-type scale ranging from 1 (*strongly disagree*) to 6 (*strongly agree*) and included “They (i.e., people in my immediate society) have many chances to get to know other people” and “They (i.e., people in my immediate society) can choose who they interact with”. In the present study, the relational mobility scale score had an internal reliability (alpha) of 0.84.

The second phase took place in the laboratory. Participants were randomly assigned to either the monitoring condition or the control condition upon arrival. In the monitoring condition, a surveillance camera was mounted in the corner of a 16ft-by-20ft room facing the participants at a 45-degree angle. Participants assigned to this condition were told that the room was previously used for research requiring monitoring and observation of participants through closed circuit television (CCTV). Participants in the control condition completed the experiment in the same room with surveillance equipment removed.

Participants were seated at a desk and given a paper cup with a small hole at the top. Inside the paper cup was a six-sided die that participants were to roll by shaking the cup. The outcome of each roll can be seen by looking through the hole. The experimenter instructed participants to roll the die at least three times and report the outcome of the first roll for pay in New Taiwan dollars (NT$) (a roll of 1 = NT$0, 2 = NT$20, 3 = NT$40, 4 = NT$60, 5 = NT$80, and 6 = NT$100). This experimental procedure received the approval of the NTU psychology department ethics committee for the protection of human participants (approval number 102-1-1024) and all participants gave their written informed consent. With this task, participants can lie and benefit financially by reporting a die roll value that is better than the actual outcome of their first roll. Because the chance of rolling any number was the same (1/6 or 16.67%), the percentage of participants who got 4, 5, or 6 should be the same as the percentage of participants who got 1, 2, or 3. Thus, we assumed that cheating occurred in a group if the percentage of high roll outcomes (rolls > 4) reported by its members in aggregate was higher than the percentage of low roll outcomes (rolls < 3).

### Results

Using logistic regression analysis, we examined whether the number of participants who reported die rolls of 1 to 3 (low) versus 4 to 6 (high) differed as a function of relational mobility and social monitoring. Instead of using omnibus interaction analyses, we performed contrast analysis, which provides a powerful and clear test of strong, a priori predictions [31–32]. According to Rosenthal et al. [31], the omnibus interaction is suboptimal for detecting specific, hypothesis-driven effects. In order to perform contrast analysis, we first classified participants into either the low mobility group or the high mobility group using median split. Next, we created a focused contrast code (+1 = low mobility and low monitoring, -1 = low mobility and high monitoring, 0 = high mobility and low monitoring, 0 = high mobility and high monitoring) and regressed the dependent variable on this contrast code.

We found no main effect of mobility (*B =* .44, *SE* = .51, Wald = 0.73, *p* = .392) or monitoring (*B =* .55, *SE* = .51, Wald = 1.17, *p* = .280) on reported die rolls. More importantly, the contrast analysis was significant, *B =* .83, *SE* = .38, Wald = 4.84, *p* = .028, Exp(*B*) = 2.301, supporting our hypothesis. The latter result suggests that the rate of reporting a high die value (4 to 6) was higher among low mobility participants in the control condition compared to low mobility participants in the monitoring condition, whereas the presence of a social monitoring cue did not affect reports of low versus high die rolls among the high mobility participants. A similar pattern of results was obtained from logistic regression analysis using relational mobility as a continuous variable (omnibus interaction: *B =* .51, *SE* = .30, Wald = 2.91, *p* = .088, Exp(*B*) = 1.66). Consistent with our hypothesis, the rate of reporting a high die value (4 to 6) was higher among low mobility participants in the control condition compared to low mobility participants in the monitoring condition, *B =* -.84, *SE* = .43, Wald = 3.91, *p* = .048, Exp(*B*) = .43. In contrast, the presence of a social monitoring cue did not affect reports of low versus high die rolls among the high mobility participants, *B =* .17, *SE* = .38, Wald = .21, *p* = .644, Exp(*B*) = 1.19.

Together, the results of Study 2 supported our hypothesis that the presence of a social monitoring cue is effective in deterring negative behavior when relational mobility is low but not when relational mobility is high. However, the quasi-experimental nature of our study precluded any causal inferences regarding the role of relational mobility in moderating the link between social monitoring and dishonesty. To further test the notion that social monitoring cues matter in reducing socially deviant behavior when individuals perceive their immediate social environment to be low (as opposed to high) in relational mobility, we conducted Study 3 in which perceptions of relational mobility were primed.

## Study 3

Because low and high relational mobility individuals were found to be differentially influenced by social monitoring in Study 2, we reasoned that priming relational mobility should produce a similar pattern of results. Instead of completing the die-under-cup task, participants in Study 3 played an online maze game that provided them an opportunity to win extra cash for the number of mazes they indicated as solved within the time period allotted. As in Study 2, we hypothesized that the presence of social monitoring cues would reduce the likelihood of cheating when relational mobility is low but not when relational mobility is high.

### Method

#### Participants

One hundred and fifty-five students (75 females, 79 males. 1 unknown; average age = 20.07 years) from NTU participated in the study for course credit. We used the most conservative estimate from previous studies on social monitoring, *d* = 0.485, to calculate the sample size we would need to have a statistical power of .80. Based on our estimate, we aimed to obtain data from at least 136 participants and stopped at the end of the semester, yielding a final sample size of 155.

#### Procedure

Participants were randomly assigned to one of two monitoring conditions (monitoring vs. control) and one of two mobility conditions (low mobility vs. high mobility). In the monitoring condition, participants completed the experimental tasks in a room equipped with a closed-circuit TV, which they were told was used previously in research requiring monitoring and observation of participants. Participants assigned to the control condition completed the experimental tasks in an identical room without a closed-circuit TV.

In order to manipulate relational mobility, we asked participants to write a persuasive essay on an assigned topic for 15 minutes. Participants in the low mobility condition were asked to generate three arguments and two personal experiences that supported the following statement: “*In our society*, *people have few opportunities and many constraints for developing new relationships*. *Moreover*, *it is fairly difficult for people to leave relationships they no longer need or want to have*”. Participants in the high mobility condition were asked to generate three arguments and two personal experiences that supported the following statement: “*In our society*, *people have many opportunities and few constraints for developing new relationships*. *Moreover*, *it is fairly easy for people to leave relationships they no longer need or want to have*”.

To ensure that the relational mobility manipulation would work as intended, we conducted a pilot study with 97 NTU students (41 females, 56 males; average age = 19.28 years). After writing the mobility priming essay, participants completed the Relational Mobility Scale [30]. Results indicated that the mobility manipulation worked as intended, with those in the high mobility priming condition scoring higher on the Relational Mobility Scale (*M* = 4.83, *SD* = 0.70) than those in the low mobility priming condition (*M* = 4.56, *SD* = 0.62), *t*(95) = 1.99, *p* = .046, *d* = .41, 95% CI = [-.537, -.005].

Following the mobility priming task, participants completed an online maze game that gave them the opportunity to earn five New Taiwan dollars for every maze solved. Each participant had a total of 30 minutes to solve as many mazes as possible using only arrows on the keyboard. Following an experimental design used by Schwieren and Weichselbaumer [33], a spy-ware program was used to collect information about participants’ actual behavior in order to identify different types of cheating. Other than to start the clock and to start new mazes, the use of any function key (“e.g., “Auto-Solve”, “Path-Verify”) was prohibited. The program also allowed us to determine the number of mazes our participants actually solved and whether they continued working after time expired. We compared these data to participants’ reporting of the number of mazes they solved to gauge whether cheating occurred.

Before starting the game, participants practiced solving an easy maze (difficulty level 1, out of 5) to familiarize themselves with the game. When ready to begin, the experimenter set the difficulty level to 4 and instructed participants to work at this level throughout the game. Participants recorded the time taken to finish each maze into a table. After completing the online maze game, participants filled out a short demographics questionnaire, received payment according to the number of mazes they reported having solved, and went through a full debriefing. After data collection was complete, a research assistant blind to our research hypothesis analyzed the spy-ware recordings to identify different types of cheating, including participants who: 1) reported having solved more mazes than they actually did, 2) used function keys to simplify or speed up the solution, and 3) worked past the time limit and counted the mazes solved overtime towards their total score. This experimental procedure received the approval of the NTU psychology department ethics committee for the protection of human participants (approval number 101-1-1012) and all participants gave their written informed consent prior to starting the experiment.

### Results

Table 1 reports the number of mazes participants actually solved, the number of mazes they indicated as solved, and the frequency of various cheating behaviors by condition. There was no difference between the low and high mobility conditions or between the monitoring and control conditions in the number of mazes solved (mobility: *t*[153] = 0.67, *p* = 0.505, 95% CI [-.934, 1,890]; monitoring: *t*[153] = 1.12, *p* = 0.266, 95% CI [-.612, 2.204]) or indicated as solved (mobility: *t*[153] = 1.25, *p* = 0.212, 95% CI [-.509, 2.278]; monitoring: *t*[153] = 1.36, *p* = 0.176, 95% CI [-.435, 2.349]).

**Table 1:** Descriptive statistics of mazes indicated, mazes solved, and cheating behavior as a function of relational mobility and social monitoring.

Variables	Low relational mobility						High relational mobility					
	All (*N* = 79)		Monitoring (*N* = 40)		Control (*N* = 39)		All (*N* = 76)		Monitoring (*N* = 37)		Control (*N* = 39)	
	Mean	SD	Mean	SD	Mean	SD	Mean	SD	Mean	SD	Mean	SD
Maze indicated	14.25	4.51	14.05	4.35	14.46	4.72	13.37	4.26	12.57	4.78	14.13	3.60
Maze solved	13.56	4.60	13.50	4.40	13.62	4.85	13.08	4.29	12.30	4.80	13.82	3.66
*Cheating* (# of mazes indicated minus # of mazes solved)	0.70	1.82	0.55	1.11	0.85	2.35	0.29	0.78	0.27	0.87	0.31	0.69
	Yes	No	Yes	No	Yes	No	Yes	No	Yes	No	Yes	No
Used function Keys	4	75	2	38	2	37	2	74	1	36	1	38
Reported mazes solved over time limit	15	64	4	36	11	28	8	68	33	4	35	4

To test our hypothesis that cheating behavior would decrease significantly from low to high monitoring when mobility is low but not when mobility is high, we created a hypothesis-specific, focused contrast code (+1 = low mobility and low monitoring, -1 = low mobility and high monitoring, 0 = high mobility and low monitoring, 0 = high mobility and high monitoring) and regressed the dependent variables on this contrast code. First, we examined whether participants counted the mazes they solved after the timer went off towards their total score. Even though none of the main effects reached statistical significance (mobility: *B =* -.69, *SE* = .47, Wald = 2.14, *p* = .144, 95% CI [-1.778, .289]; monitoring: *B =* -.72, *SE* = .47, Wald = 2.33, *p* = .127, 95% CI [-1.919, .164]), the contrast analysis was statistically significant, *B =* .75, *SE* = .34, Wald = 4.90, *p* = .027, Exp(*B*) = 2.113, 95% CI [.109, 1.665], supporting our hypothesis that the presence of a social monitoring cue reduced cheating in the low mobility condition but not in the high mobility condition.

Next, we examined the effects of mobility and monitoring on the use of function keys. As Table 1 indicates, very few participants used the function keys to help simplify or speed up the solution. Hence, we found no main effect of mobility or monitoring on this type of cheating behavior (mobility: *B =* -.68, *SE* = .88, Wald = .56, *p* = .441, 95% CI [-18.677, 1.360]; monitoring: *B =* .01, *SE* = .83, Wald = .00, *p* = .987, 95% CI [-17.997, 17.984]) and the result of the contrast analysis was not statistically significant (*B =* .01, *SE* = .58, Wald = .00, *p* = .982, 95% CI [-1.781, 1.763]). With respect to the difference in number of mazes solved and number of mazes indicated as solved, participants in the low mobility condition over-reported more than those in the high mobility condition on a marginally significant level, *t*(107) = 1.82, *p* = 0.072, *d* = .293, 95% CI [-.037, .850]. Moreover, even though the social monitoring cue had a greater impact on deterring cheating in the low mobility condition compared to the high mobility condition, the difference did not reach statistical significance, *B =* .15, *SE* = .16, *t* = .91, *F*(1, 153) = 0.83, *p* = .364, 95% CI [-.190, .631].

## Discussion

Does residential and relational stability heighten individuals’ sensitivity to social monitoring? To answer this question, we examined the impact of social monitoring on deviant behavior at both the community level (Study 1) and the individual level (Studies 2 and 3). Across the three studies, we found that social monitoring exerted greater influence on curbing social deviance in more stable environments. Although avoiding being identified as a norm violator is a major concern for humans in general, monitoring of reputational information may be greater when moving around is a more complicated and costly affair.

Previous research has suggested that sensitivity to reputational information plays a key role in human cooperation [5–6], as well as in the deterrence of deviance [34–36]. However, our studies show that the power of social monitoring on social deviance can vary according to social-structural constraints such as residential mobility and relational mobility. In contexts that are similar to our evolutionary ancestors, i.e., low in residential or relational mobility [37–38], social monitoring may be an effective mechanism of social control. People’s willingness to monitor each other’s merits as well as misdeeds may play a crucial role in the functioning of residentially and relationally stable societies. By contrast, the effects of social monitoring and the appeal to reputation might not be as strong in residentially and relationally mobile contexts. Our results are consistent with recent findings on rural-urban differences in interpersonal regret [11]. The thought of rural living evoked greater reputational concern, which in turn increased the intensity of interpersonal regret, relative to the thought of urban living. Together these data suggest that the monitoring of reputation as a mechanism of informal social control may be stronger in smaller, residentially/relationally stable communities than in larger, residentially/relationally mobile communities where there is always an exit option if one’s reputation is damaged.

In the present research, we focused on just one mechanism of social control. Exploration of alternative mechanisms would be worthwhile, particularly those that can shed light on how social order is effectively maintained in residentially or relationally mobile contexts. In societies where individuals change groups frequently, continuously attending and adjusting to new group norms and regulations can be difficult and taxing. Therefore, we propose that members from mobile societies are more apt to follow universal rules of conduct (e.g., law) and to develop an independent, internal code of ethics. This notion is consistent with cultural psychological theories and research on rights-based vs. duty-based moral codes [39–40]. Specifically, the moral judgments and behaviors of individuals living in the US, which is a highly mobile society, are guided by personal preferences [41] and individually-oriented moral codes [40]. Future studies examining whether social control in residentially or relationally mobile settings can be achieved via internalized moral values and standards hold promise in advancing this area of research.

It is worth noting a few limitations of the study. First, convenience samples of undergraduate students comprise a narrow database, creating problems for the generalizability of our findings to larger populations of interest [42–44]. Second, we relied on official crime statistics from a government security agency, which may not accurately reflect crime rates in a given community due to issues such as under-reporting and under-recording [45–48]. Third, even though the power of the news media in shaping a person’s reputation has been well documented [22–23] and that its role in shaming people when they commit minor offences has intensified in recent years [49], the validity of using number journalists employed as a measure of the prevalence of social monitoring needs to be further established. The work of Baum and Potter [50] suggests that the media plays a critical role in controlling the flow of information from whistle blowers to the public. Finally, sample sizes for Studies 2 and 3 were small, making interpretations of the findings more difficult. The reliability of the key finding (i.e., social monitoring deters deviant behavior in low mobility contexts) needs to be examined using larger samples in future research, especially since the pattern was observed with only some of the cheating indices. Aside from the issue of reliability, possible floor effects involving cheating behaviors that were too obvious and difficult to justify could have contributed to the null findings. Previous studies have shown that people are more willing to cheat when they can justify their unethical behavior [28, 51]. For example, participants in Study 3 may have felt that counting mazes solved after the time limit towards their total score was legitimate, given that they actually did solve them on their own (as opposed to reporting ones they never solved). In contrast, using function keys and reporting mazes they never saw are obvious, difficult-to-justify lies, which might explain why very few participants engaged in these forms of cheating and thus creating floor effects that made the effects of social monitoring on cheating hard to detect.

As human societies become more interconnected, issues related to trust and cooperation among individuals and groups deserve greater attention than ever before. Any viable solution to the problems of crime, deception, and mistrust may benefit from considering particular features of the environment in which individuals and groups inhabit. Recent perspectives in cultural psychology—including the dynamic constructivist approach [52], the institutional approach [53], the intersubjective approach [54], and the socio-ecological approach [55]—have highlighted the importance of the social ecological environment in shaping humans’ thoughts, feelings, and behavior. By examining how residential and relational mobility influence individuals’ reaction to cues of social monitoring, we hope to facilitate a potentially fruitful avenue for future research on the psychology of social cooperation and deviance.
